# Bibliometric analysis of the trends in research on the link between gut microbiota and pancreatic cancer

**DOI:** 10.3389/fcimb.2026.1623945

**Published:** 2026-03-02

**Authors:** Zhimin Yu, Shixia Cai, Zhantao Shen, Guihao Chen, Yanchen Chen, Yifeng Liu, Xiaosheng Zhong

**Affiliations:** Pancreas Center, Guangdong Provincial Hospital of Traditional Chinese Medicine, The Second Affiliated Hospital of Guangzhou University of Chinese Medicine, Guangzhou, China

**Keywords:** bibliometric analysis, gut microbiota, hotspots, pancreatic cancer, research trends

## Abstract

**Background:**

Pancreatic cancer (PC) is a highly prevalent and aggressive malignancy of the digestive system, characterized by notably low survival rates. Recently, the influence of gut microbiota (GM) on the development and progression of PC has gained more attention. While the only existing bibliometric study has explored the association of GM in PC, it has failed to adequately reflect the latest trends and hotspots in this field due to research timeframes and methodologies.

**Methods:**

Publications from January 1, 2000 to November 18, 2023 were collected from the Web of Science Core Collection (WoSCC) and screened based on specific criteria. Various software tools, including VOSviewer, Scimago Graphica, R, Pajek64 and Cite Space, were employed in this bibliometric study to visualize research trends and hot spots concerning the relationship between GM and PC.

**Results:**

This study analyzed 763 publications, including 397 articles and 366 reviews, on the relationship between GM and PC. The number of Publications has steadily increased since 2013, with China and the USA leading in contribution. The journal *Cancers* published the most papers (37), while *Gut* had the highest average citations (136.83). The most productive institution was SUTMD Anderson Cancer Center, and the top 3 authors were Michaud, Dominique S. McAllister, Florencia, and Miller, George. Keyword analysis revealed that “Gut Microbiota”, “Pancreatic Cancer”, “Cancer”, “Sequence”, “Microbiome” and “Tumor Microbiome” are the most frequent terms, highlighting key research trends.

**Conclusions:**

Over the past two decades, interest in the relationship between GM and PC has significantly increased. The comprehensive bibliometric analysis offers an in-depth evaluation of the prevailing research trends and advancements regarding the relationship between GM and PC. It indicates that current research hotspots mainly focus on “sequencing”, “microbiomes” and “tumor microbiomes”.

## Introduction

Pancreatic cancer (PC) is a prevalent and highly aggressive malignancy of the digestive system, with a dismal 5-year survival rate of merely 11% (Khalaf et al., 2021). While various risk factors—such as age, obesity, genetic predisposition, alcohol use, smoking, chronic pancreatitis, and diabetes—have been identified, the exact etiology of PC remains unclear (Klein, 2021; Zanini et al., 2021; Bennett et al., 2022; Amri et al., 2023; Michalak and Malecka-Wojciesko, 2023; Conway et al., 2024). Owing to its subtle early symptoms and the absence of specific detection indicators, PC is often not diagnosed until advanced stages. Consequently, research has increasingly focused on early diagnosis, tumorigenesis mechanisms, and anti-tumor therapy.

The gut microbiota (GM), comprising trillions of microorganisms, exhibits a complex relationship with cancer (Aykut et al., 2019; Dong et al., 2021; Hashimoto et al., 2023; Seo and Wargo, 2023). Advances in targeted 16S rRNA pyrophosphate sequencing and metagenomics have shown that abnormal GM contribute to chronic metabolic diseases like chronic pancreatitis, obesity, inflammatory bowel disease (IBD), and nonalcoholic fatty liver disease (NAFLD), as well as tumors (Jandhyala et al., 2015; Jandhyala et al., 2017; Riquelme et al., 2019; Talukdar et al., 2021; Zhao et al., 2022; Feng et al., 2023; Ma et al., 2023). Emerging evidence indicates that gut dysbiosis is significantly linked to PC (Yu et al., 2022; Chen T. et al., 2023). Researchers have identified several mechanisms by which the GM influences PC, including modulation of the microbe-immune system tumor axis, alteration of the tumor microenvironment, impact on metabolite production, promotion of cancer-related inflammation, and effects on chemotherapy efficacy (Aykut et al., 2019; Dong et al., 2021; Li et al., 2022; Zhang and Tang, 2022; Hashimoto et al., 2023; Seo and Wargo, 2023). Obviously, these findings highlight the complex relationship between GM and PC, functioning as a carcinogenic factor while also representing a promising therapeutic target (Kartal et al., 2022).

Bibliometric analysis has emerged as a key method for assessing the credibility, quality, and impact of scholarly work by analyzing and visualizing research trends in the era of big data (Jiang et al., 2022; Mirhashemi et al., 2022; Sun et al., 2022). Unlike traditional systematic reviews, bibliometric analysis offers a quantitative approach to assess academic literature, enabling the identification of key research findings, contextual development, and summarization of research trends (Kokol, 2021; Ge et al., 2022). To the best of our knowledge, only one study has quantitatively analyzed the literature on the relationship between GM and PC (Wu et al., 2023). However, previous study failed to adequately reflect the current research status and hotspots in the relationship between GM and PC. Constrained by research timelines and methodological limitations, some pivotal and emerging studies may have been omitted in the previous study. Furthermore, the exclusion of investigational hotspots that lack established feasibility would compromise the comprehensiveness of emerging trends mapping.

In contrast, this study incorporated data from the most recent years (updated to November, 2023). In an era defined by the rapid evolution of big data, one-year new data may lead to groundbreaking high-impact papers, new research directions, and shifts in the landscape of leading countries and institutions. Furthermore, our study covered a longer time span (dating back to 2000), allowing us to better analyze the evolution of research hotspots and future direction rather than merely focusing on current trends. By refining our search terms, we have generated a literature set that is more focused on the core relationship between GM and PC, thereby enhancing the accuracy and relevance of our analysis. This approach would more effectively reveal the “historical patterns” of development in the field.

## Methods

### Search strategy

In this study, the Web of Science (Core Collection) served as the primary database. We collected relevant literature based on titles (TI) and abstracts (AB) using the following search strategy: ((((ALL=(gut OR intestine* OR gastrointestin* OR bowel OR Gastrointestinal Tracts OR GI Tract OR GI Tracts OR Digestive Tract OR Digestive Tracts)) AND ALL=(microbiome OR microbiota OR microorganism OR microbe OR microbiota* OR microbiome* OR flora* OR microflora* OR microecology OR 16Sr* OR metagenome OR bacteria* OR prebiotic* OR probiotic* (Chen H. et al., 2023) OR antibiotic* OR dysbiosis* or Saccharomyces* OR Lactobacillus* OR Bifidobacterium* OR Escherichia coli*)) AND ALL=(Pancreas* Neoplasms OR Neoplasm* Pancreas OR Neoplasm* Pancreas* OR Pancreas* Neoplasm* OR Neoplasm* Pancreatic* OR Cancer* of Pancreas OR Pancreas* Cancers OR Pancreas* Cancer* OR Cancer* Pancreas OR Cancer* Pancreatic* OR Cancer* Pancreas* OR Pancreatic* Cancer* OR Cancer* Pancreatic OR Cancer* Pancreatic* OR Pancreatic* Cancers OR Cancer* of the Pancreas OR Pancreatic* Ductal Adenocarcinoma*)) AND LA=(English)) AND DT=(Article OR Review).

### Study selection

**Figure 1:**
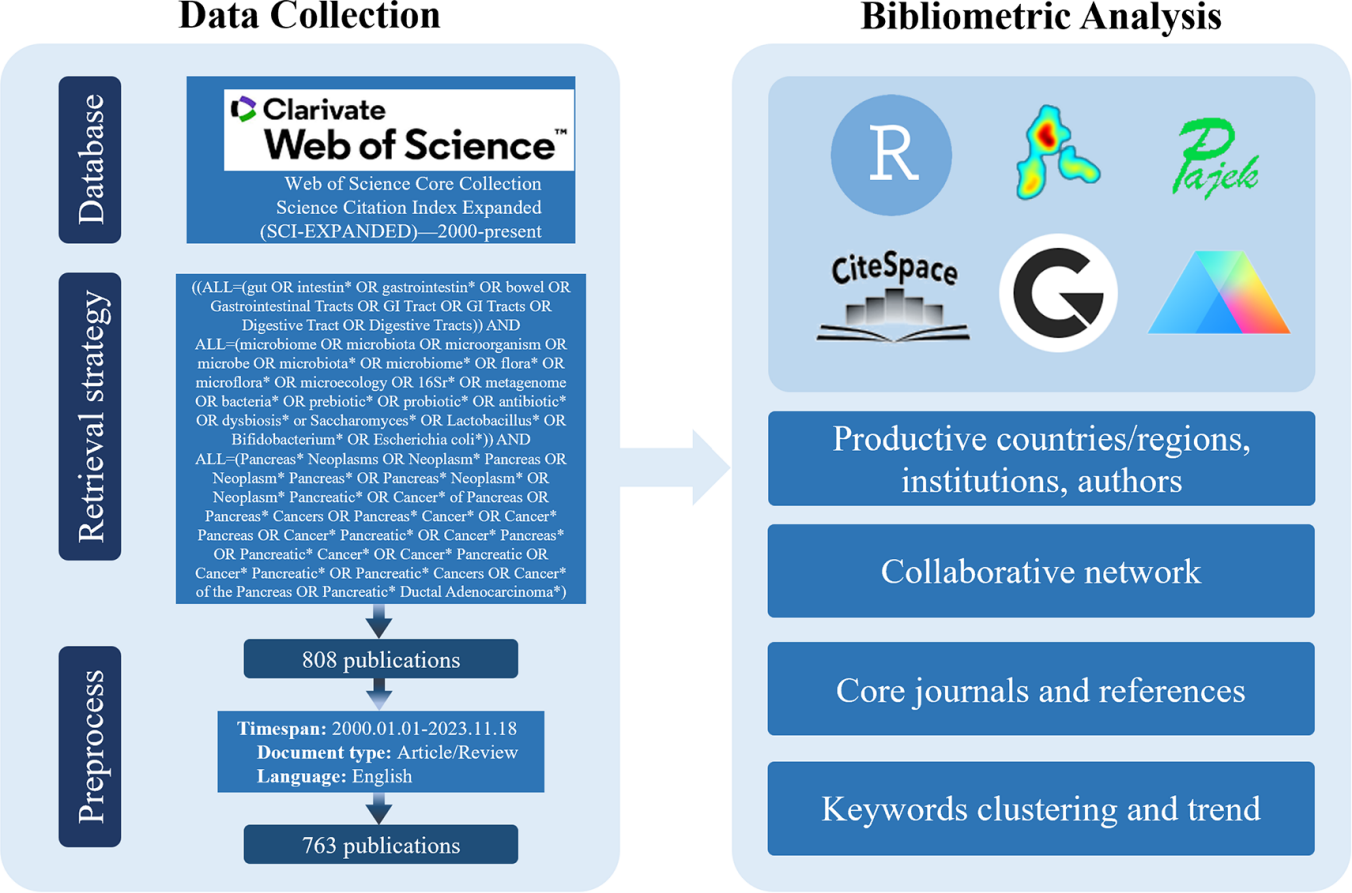
Retrieval and analysis flowchart.

The selection criteria and literature screening process for this study are shown in **Figure 1**. We began with an initial search using specific terms, followed by two researchers reviewing the identified publications. We excluded those that did not meet the following criteria: (1) publications must be in English; (2) only articles and reviews were included, excluding letters, comments, or conference abstracts; (3) sources had to come from the WoSCC Citation Index Expanded (SCI-E) and Social Sciences Citation Index (SSCI); (4) the publication period was from January 1, 2000, to November 18, 2023; (5) selected studies needed to involve PC patients, animal models, or cellular models, and assess the correlation with gut microbiota. To prevent bias from daily database updates, two researchers independently conducted the initial screening on the same day. Any discrepancies were addressed through a consensus-based discussion. Any remaining disputes were settled by a third investigator.

### Data acquisition

The articles retrieved from the WOS search were exported in various formats for further analysis, extracting indicators such as the number of publications, citation frequency, country, institution, journal, author, keyword, 2022 journal impact factor, and H-index (the number of papers with citations greater than or equal to H).

### Data analysis

Before analysis, we grouped Wales, Northern Ireland, Scotland, and England as the UK. We used VOSviewer (version 1.6.19), CiteSpace (version 6.1R6), Scimago Graphica Beta 1.0.3, and the bibliometrix package (version 4.1.2) based on R (version 4.3.1) for bibliometric and visualization analysis. VOSviewer, Pajek64, and Scimago Graphica were employed to create knowledge maps of influential authors, countries, institutions, core journals, high-quality papers, co-occurring keywords, and co-cited references. VOSviewer was employed to identify the keywords with the highest frequency of occurrence, analyze bibliometric coupling among journals, and cluster the top 54 keywords. In VOSviewer visualizations, each node typically represents a country, organization, or author. The size of a node is proportional to its publication output, and the thickness of the connecting lines (links) reflects the strength of collaboration between entities. To generate a more intuitive geographic distribution map, Scimago Graphica was utilized with data sourced from VOSviewer. In the map, point size represents the number of publications, with the width of the connecting lines denoting the level of collaboration between countries (Huang et al., 2023). Additionally, CiteSpace was utilized to extract keywords and references from highly cited publications and generate a dual-map overlay illustrating the connections between journals associated with GM and PC. Furthermore, CiteSpace assessed the level of collaborative centrality among countries/regions, institutions and authors. Subsequently, trend topic detection within the bibliometric program was implemented as an alternative methodology. Due to the absence of a consensus regarding the optimal bibliometric analysis approach, an integration of their respective properties and advantages was undertaken.

## Results

### Analysis of publications in continents/regions over the years

**Figure 2:**
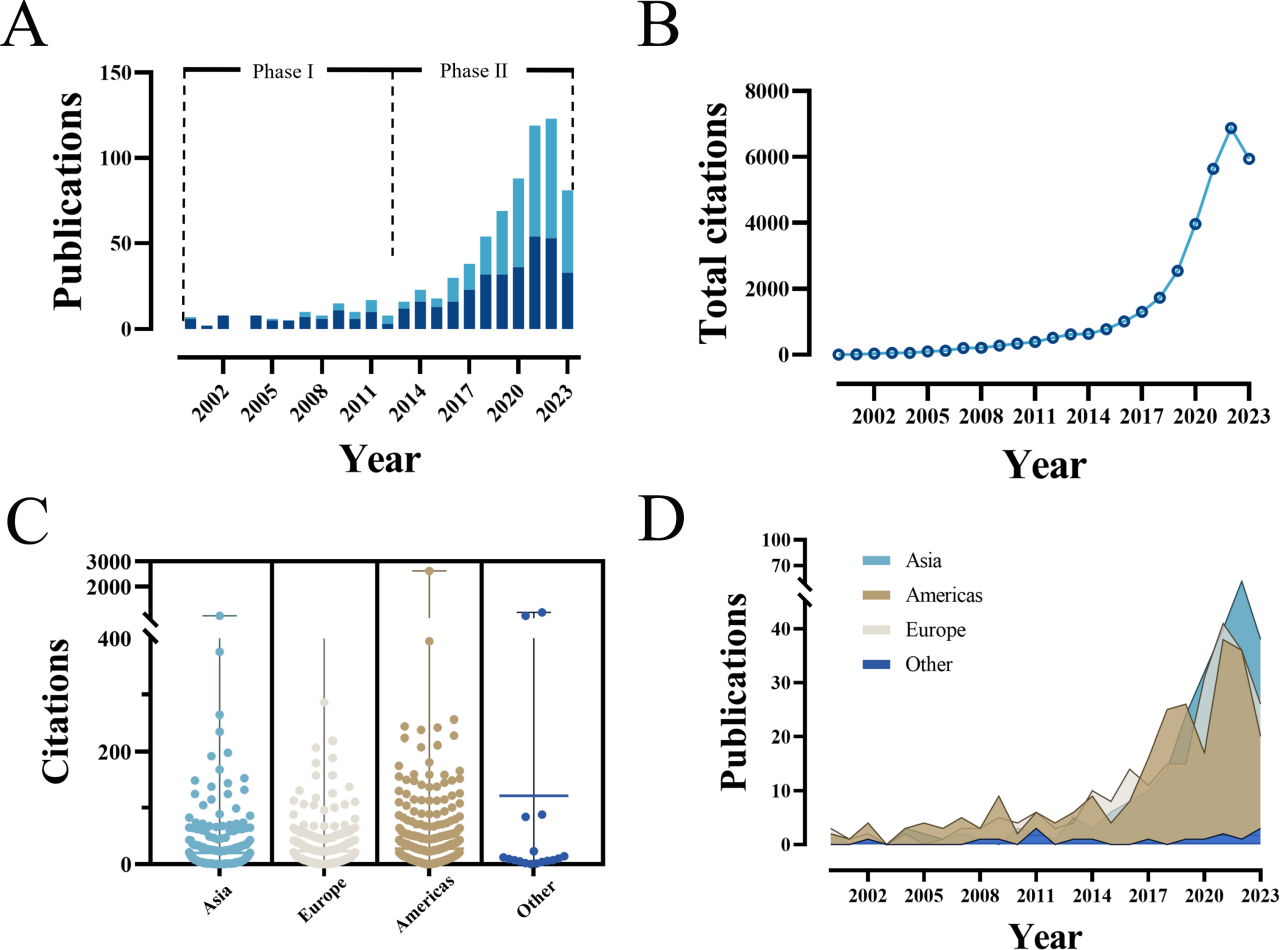
Overview of literatures over the years and continents. **(A)** The global annual publications during initial and rapid growth periods. **(B)** The total citation count of publications increases year by year. **(C)** The number of citations attributed to each continent/region. **(D)** The annual number of global publications by continent/region. ζ: Other: Oceania and Africa.

A total of 763 documents, comprising 397 articles and 366 reviews, from January 1, 2000 to November 18, 2023 were enrolled in for bibliometric analysis after screened by the inclusion and exclusion criteria in this study (**Figure 1**). The annual number of publications has significantly increased over the past 23 years, particularly in the last decade, peaking at 123 in 2022 (**Figure 2A**). Besides that, the number of citations has been gradually increasing from 2000 to 2023. Since 2014, the number of citations has obtained rapid progress, and it has reached a high value (over 6000 total citations) in 2021 (**Figure 2B**). Additionally, in terms of continental/regional distribution, the number of publications in the Americas and Asia has increased remarkably since 2017 (**Figure 2D**). Moreover, the literature originating from the Americas and Asia achieved the highest citation counts. (**Figure 2C**). These findings indicate that the link between GM and PC has gained increasing attention in recent years, and may emerge as a global hotspot of future research.

### Countries/regions analysis

**Figure 3:**
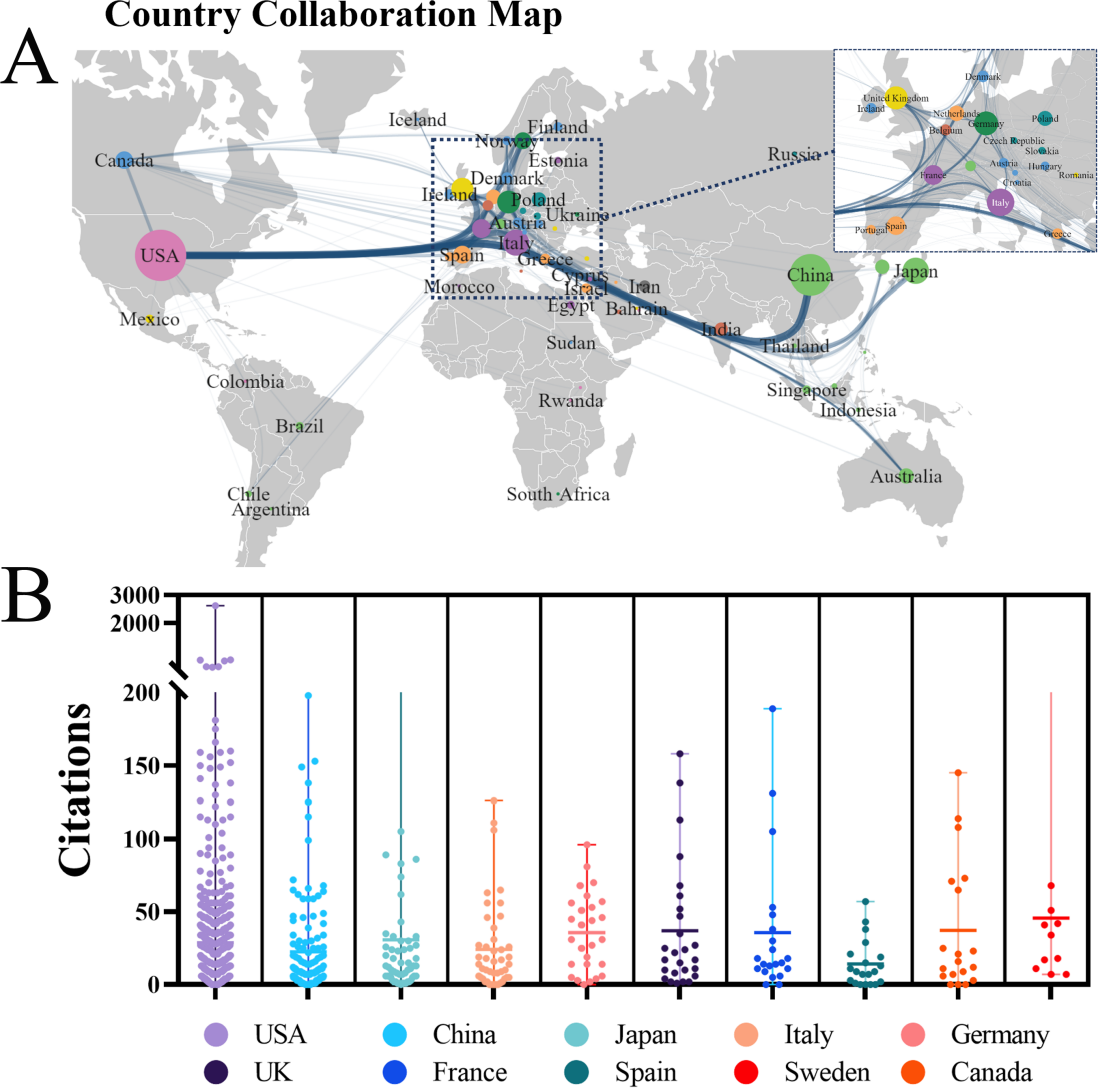
Analysis of international collaboration and literature citation. **(A)**. The cooperation network of countries/regions generated with R software. **(B)** Citation counts of the top 10 most productive countries.

A total of 63 countries/regions have published studies on GM and PC as shown in the network map (**Figure 3A**). China and the United States are the most central and closely connected countries. the United States has links to nearly all countries, particularly stronger ties with the UK, Japan, Italy, Germany and France. In contrast, China has fewer connections, mainly with Japan, South Korea and Canada. The cooperation network among these countries is illustrated in **Figure 3A**. **Figure 3B** depicts the citation counts of publications from the top 10 countries contributing to GM research in PC. The United States demonstrated the most substantial academic impact by total citations (18,736), far surpassing China (3,895) and Italy (2,667). When impact is normalized by publication volume, however, France achieved the highest average citation rate (109.3), followed by Sweden (77.32), the U.S. (72.34), and Canada (65.17).

### Contribution of institutions and authors

**Figure 4:**
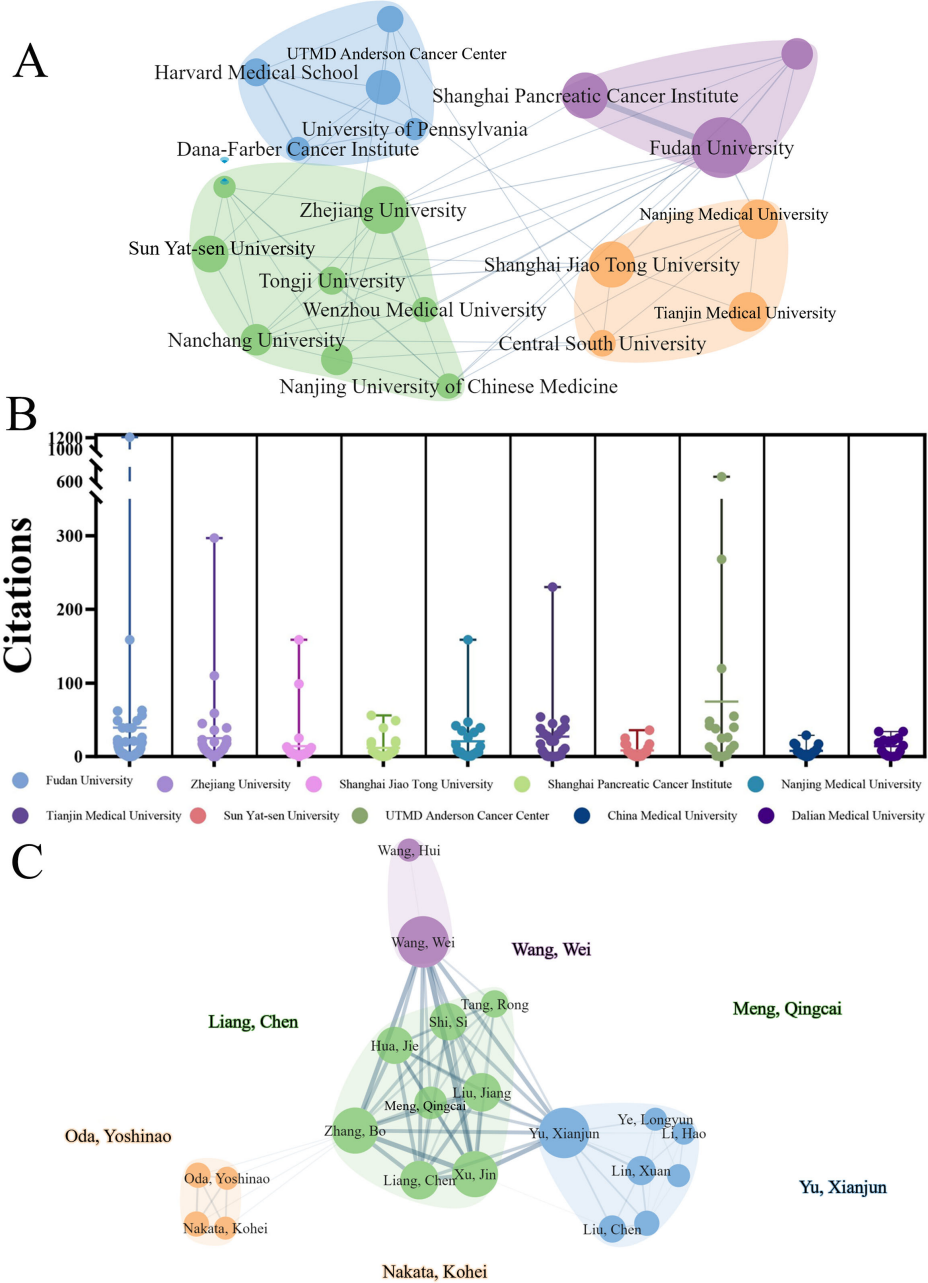
Collaborative clustering of institutions and authors. **(A)** Cooperative clustering of 25 institutions (each with over 7 publications). **(B)** Citation counts of the top 10 productive institutions. **(C)** The co-author network visualization of the top 30 authors (each with at least 4 publications) by VOSviewer.

The study assessed the most prolific universities and institutions, as shown in **Table 1**. UTMD Anderson Cancer Center produced the highest number of papers, followed by Shanghai Jiao Tong University, Zhejiang University, Karolinska Institute, and Mayo Clinic. In addition, UTMD Anderson Cancer Center had the highest total and average citations. Cooperative clustering of 25 institutions with over 7 publications was presented in this network (**Figure 4A**), where these 25 universities/institutions were classified into six groups with different colors. UTMD Anderson Cancer Center, Zhejiang University, Harvard Medical School, John Hopkins University, Baylor college of Medicine and Mayo clinic were the core nodes. In the **Figure 4B**, the number of citations from the top 10 productive institutions was presented. Simultaneously, the top 10 productive authors were also assessed in this study (**Table 2**). Professor Michaud, Dominique S from the Brown University was the most productive author with 160 publications, followed by Mcallister, Florencia(88) and Miller, George(76). Richard B. Hayes and Jiyoung Ahn shared the highest total (1,464) and average (292.8) citation counts, while Hayes alone had the highest H-index of 100. The co-authorship analysis of the 30 authors who published more than 4 papers was analyzed by VOSviewer (**Figure 4C**).

**Table 1:** The top 10 productive institutions.

Rank	Institution	Country	Publications (%)	Total citation	Average citation
1	UTMD Anderson Cancer Center	USA	18 (2.36%)	4230	235.00
2	Shanghai Jiao Tong University	China	13 (1.70%)	568	43.69
3	Zhejiang University	China	13 (1.70%)	370	28.46
4	Karolinska Institute	Sweden	12 (1.57%)	329	27.42
5	Mayo Clinic	USA	12 (1.57%)	746	62.17
6	New York University	USA	12 (1.57%)	2670	222.50
7	Peking Union Medical College	China	11 (1.44%)	142	12.91
8	Catholic University of the Sacred Heart	Italy	11 (1.44%)	290	26.36
9	Harvard Medical School	USA	10 (1.31%)	776	77.60
10	National Cancer Institute	USA	10 (1.31%)	1499	149.90

**Table 2:** Top 10 productive authors.

Rank	Author	Institution	Country	Publications (%)	Total citation	Average citation	H-index
1	Michaud, Dominique S.	Brown University	USA	160 (8.35%)	405	50.63	58
2	Mcallister, Florencia	UTMD Anderson Cancer Center	USA	88 (4.58%)	131	18.71	62
3	Miller, George	University of Central Lancashire	United Kingdom	76 (3.96%)	1036	148.00	53
4	Panebianco, Concetta	IRCCS Casa Sollievo della Sofferenza	Italy	66 (3.44%)	152	21.71	17
5	Pazienza, Valerio	IRCCS Casa Sollievo della Sofferenza	Italy	57 (2.97%)	152	21.71	36
6	Jobin, Christian	University of Florida	USA	42 (2.19%)	403	67.17	56
7	Ahn, Jiyoung	Chungbuk National University	USA	37 (1.93%)	1464	292.80	21
8	Hayes, Richard B.	NYU Grossman Sch Med	USA	37 (1.93%)	1464	292.80	100
9	Saxena, Deepak	Indian Institute of Public Health Gandhinagar	India	36 (1.88%)	471	94.20	17
10	Thomas, Ryan M.	University of Florida	USA	31 (1.62%)	259	51.80	18

### Core journals visualization analysis

**Figure 5:**
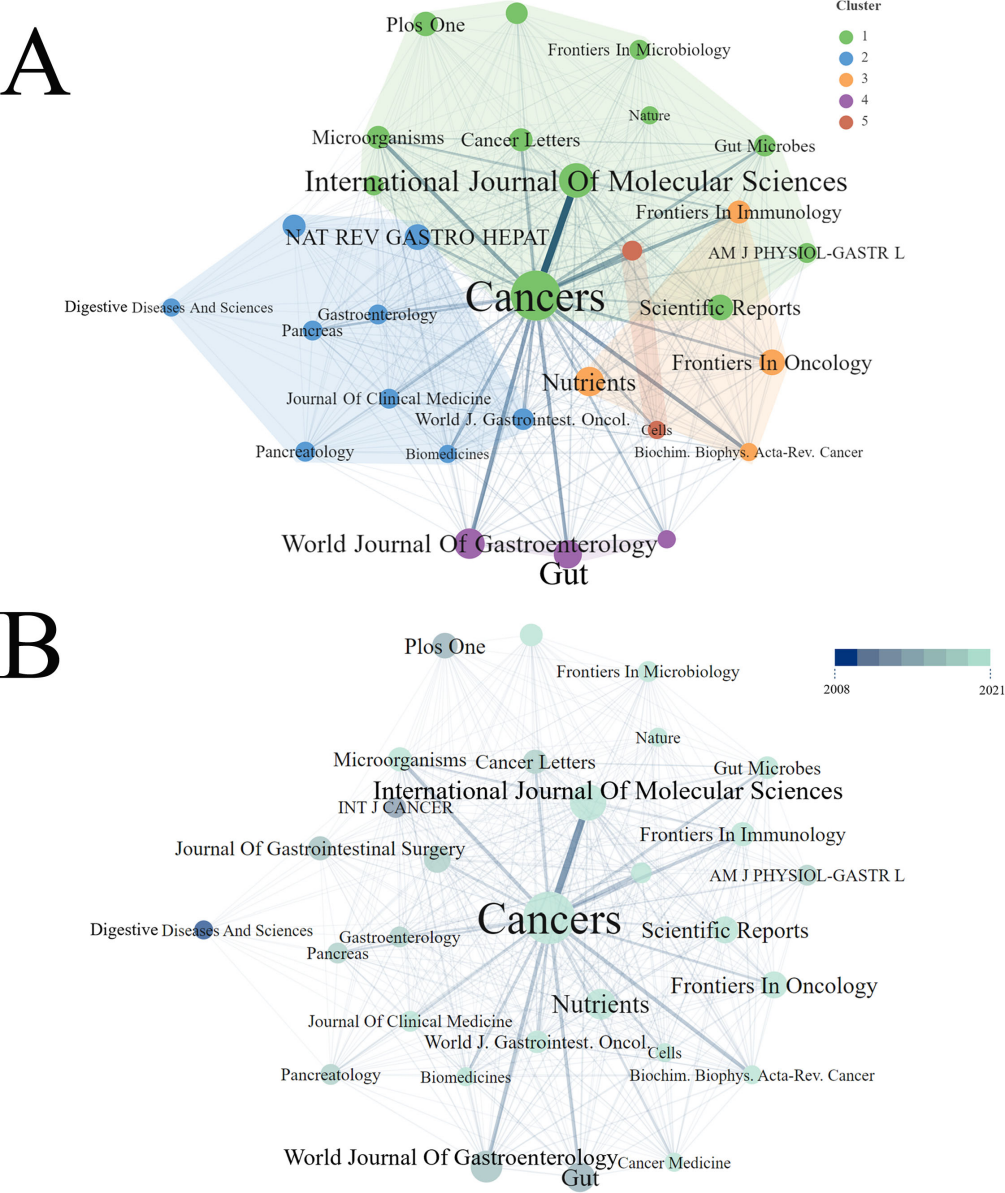
Visualization of the top 30 core journals over time. **(A)** Bibliometric coupling within top 30 journals. Five clusters were identified based on CiteSpace. **(B)** The visualization of the top 30 core journals over time. Individual node represents a Journal. The color gradient represents a timeline, with lighter shades indicating more recent years.

**Figure 6:**
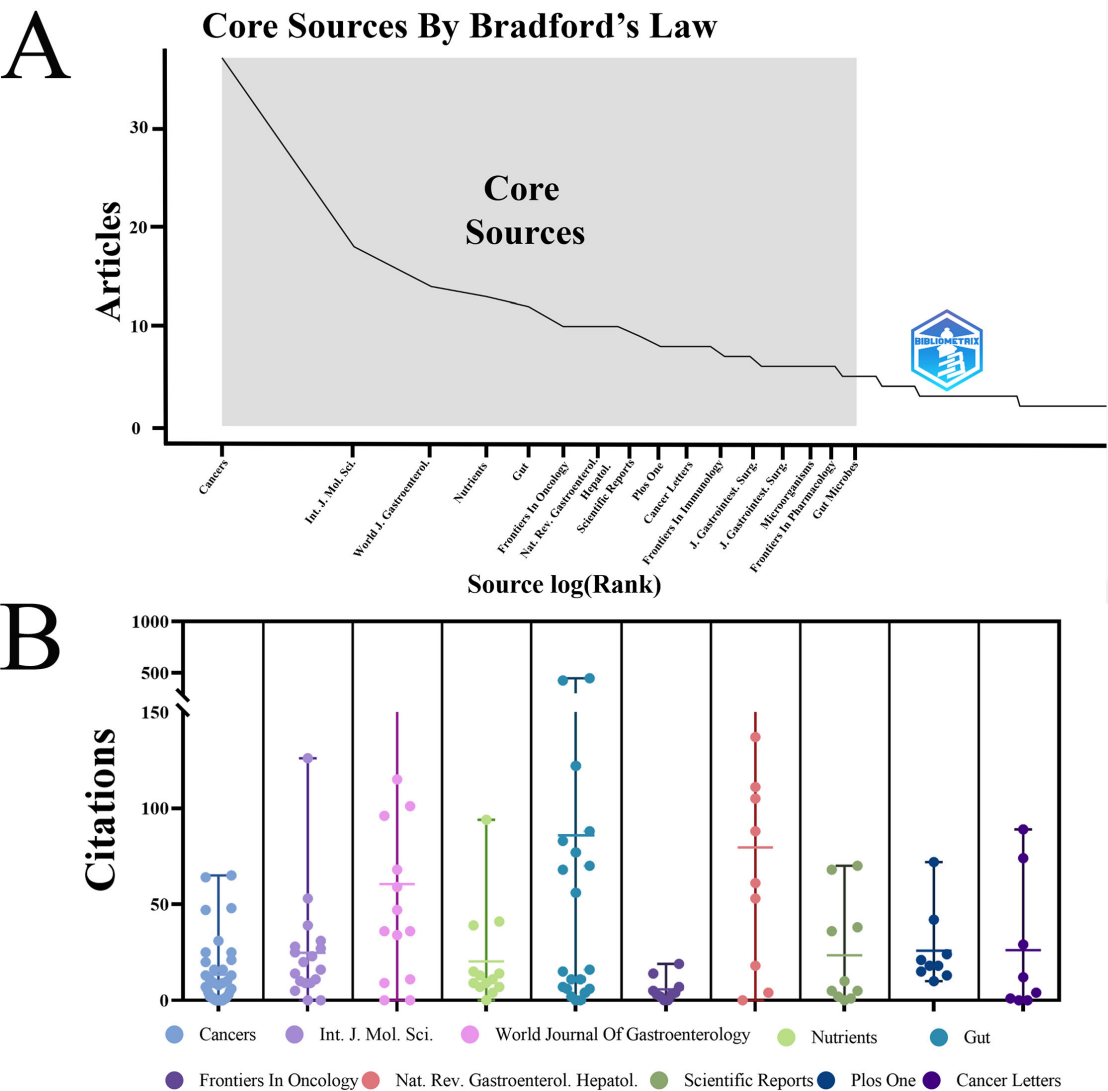
Core academic Journals and annual citation. **(A)** The 16 core academic journals identified according to Bradford’s Law using R software. **(B)** The annual citations in the 10 of top 16 core journals.

**Figure 7:**
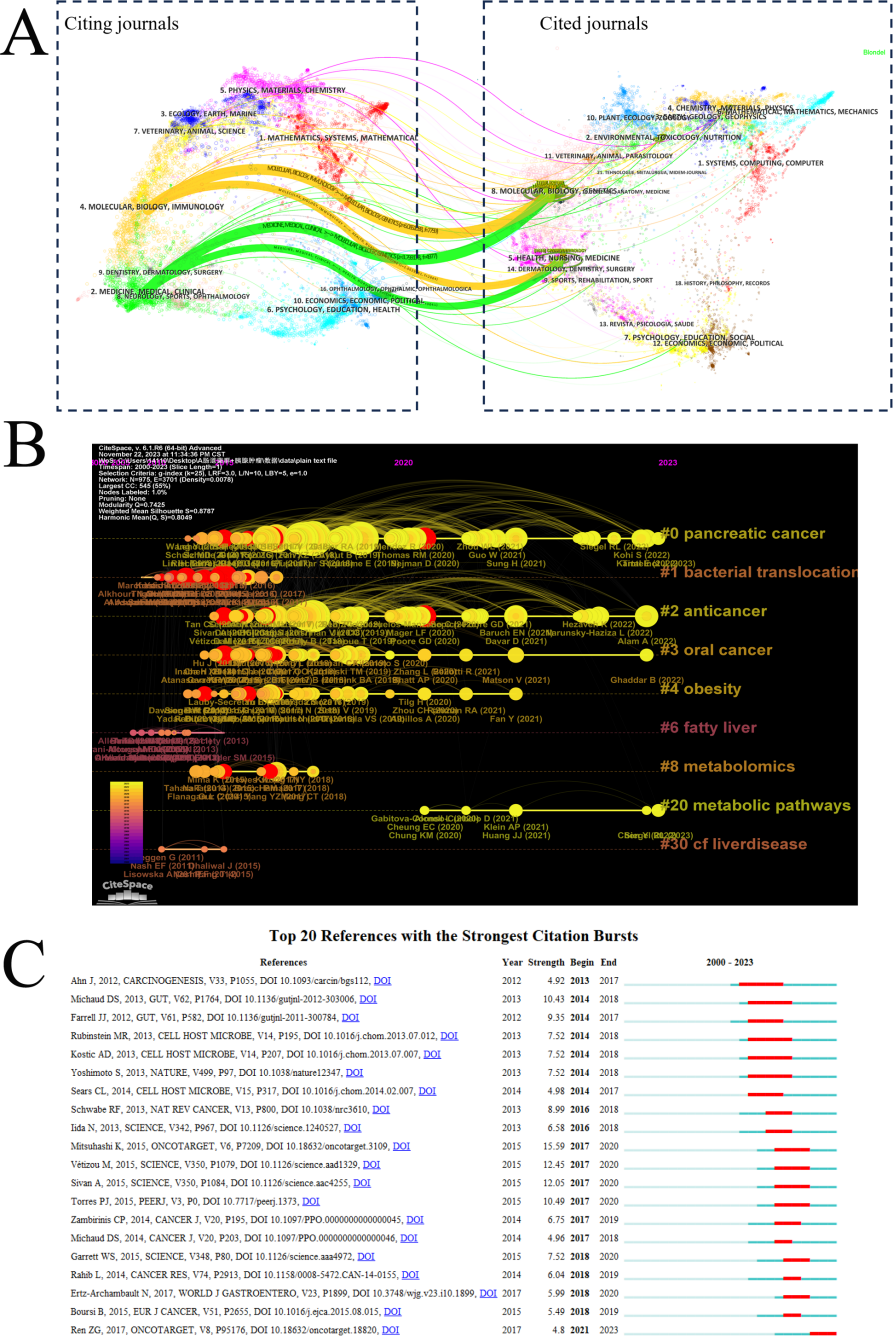
Visualization of references. **(A)** The dual-map overlay of journals related to GM-PC from 2000 to 2023 by CiteSpace. Citing journals are on the left, and cited journals are on the right. **(B)** The timeline view map of reference co-citation analysis generated by CiteSpace. The clusters ranked in descending order by size. The horizontal axis represents time, and the vertical axis represents keyword clustering. Individual node represents the cited literature, with their horizontal position indicating the time of first occurrence. Links between nodes denote co-citation relationships, and the node size is scaled according to the number of references. **(C)** The top 20 references with the strongest citation bursts. The light blue bar represents the timeline; The red bar represents the burst time period of the references.

A cooperative clustering analysis, visualized using VOSviewer, grouped the top 30 core journals (each with a minimum of five publications) into five distinct subclusters (**Figures 5A, B**). Based on the Brayford law in R software, 16 core journals were identified in the **Figure 6A**. **Table 3** summarized the top 10 core journals ranked by publications from 2000 to 2023. All the top 10 core journals were classified as Q1 or Q2 referring to Journal Citation Report 2022 criteria. The majority of publications were derived from the Cancers with 37, followed by International Journal of Molecular Science (18), World Journal of Gastroenterology (14) and other journals. Among them, the journal with the highest total citation was Gut, followed by World Journal of Gastroenterology and Nature Reviews Gastroenterology & Hepatology and other journals (**Figure 6A**). Similarly, the journal with the highest average citation was Gut, followed by Nature Reviews Gastroenterology & Hepatology and World Journal of Gastroenterology and other journals (**Figure 6B**). Notably, among these top 10 core journals, both the Gut and the Gastroenterology & Nature Reviews Gastroenterology are internationally recognized, with impact factors of 24.5 and 65.1, respectively. **Figure 7A** shows a dual-map overlay of academic journals, illustrating citation relationships between citing journals (left) and cited journals (right). It identifies two main citation paths: the orange and green paths, indicating that articles from molecular biology, genetics, health, nursing, and medicine journals are frequently cited by those from molecular biology, immunology, and clinical journals. A timeline visualization was constructed based on co-citation clustering to chronologically present the evolution of research hotspots (**Figure 7B**). The view positions older citations on the left and more recent ones on the right, with points of the same color on a line indicating the same publication year. Obviously, 1 bacterial translocation, 6 fatty liver and 8 metabolomics, located at the leftmost end of the line, were early research topics in this field. In contrast, 0 pancreatic cancer, 2 anticancer and 20 metabolic pathways were the current new research focus in this field.

**Table 3:** 10 Core journals.

Rank	Journal	Publications	Total citations	Average citations	2022 JCR category quartile	2022 IF
1	Cancers	37	478	12.92	Q1	5.2
2	International Journal Of Molecular Sciences	18	430	23.89	Q1	5.6
3	World Journal Of Gastroenterology	14	813	58.07	Q2	4.3
4	Nutrients	13	250	19.23	Q1	5.9
5	Gut	12	1642	136.83	Q1	24.5
6	Frontiers In Oncology	10	55	5.50	Q2	4.7
7	Nature Reviews Gastroenterology & Hepatology	10	760	76.00	Q1	65.1
8	Scientific Reports	10	227	22.70	Q2	4.6
9	Plos One	9	222	24.67	Q2	3.7
10	Cancer Letters	8	198	24.75	Q1	9.7

### Core literature analysis

The top ten core literature with the highest citations of GM in PC domain among the 763 publications were listed in **Table 4**. Among them, the most cited article was Gopalakrishnan, V et al (Gopalakrishnan et al., 2018) published in Science in 2018 with 2622 citations. This article illustrated that gut microbiome may modulate responses to anti PD-1 immunotherapy in melanoma patients, uncovering the microbiome’s impact on immune checkpoint blockade and paving the way for subsequent research into gut microbiota in PC. The second and third cited article were published by McGuckin, Michael A.et al (McGuckin et al., 2011) and Linden, S. K. et al (Linden et al., 2008), receiving 966, 830 citations, respectively. These two publications primarily explored the interactions between intestinal pathogens and mucins. Such interactions offer valuable insights for subsequent research on the role of GM in PC immunotherapy. Additionally, study conducted by Pushalkar et al (Chen H. et al., 2023). dedicated to investigating the microbiome’s influence on PC development and prognosis. These publications demonstrated that GM plays a key role in tumorigenesis, progression and immunotherapy of PC.

**Table 4:** 10 core literatures with the highest citations on the association between gut microbiota and pancreatic cancer.

Rank	First author	Title	Journal	Type	Year of publication	Total citations
1	Gopalakrishnan, V.	Gut microbiome modulates response to anti-PD-1 immunotherapy in melanoma patients	Science	Article	2018	2622
2	McGuckin, Michael A.	Mucin dynamics and enteric pathogens	Nature Reviews Microbiology	Review	2011	966
3	Linden, S. K.	Mucins in the mucosal barrier to infection	Mucosal Immunology	Review	2008	830
4	Nejman, Deborah	The human tumor microbiome is composed of tumor type-specific intracellular bacteria	Science	Article	2020	829
5	Pushalkar, Smruti	The Pancreatic Cancer Microbiome Promotes Oncogenesis by Induction of Innate and Adaptive Immune Suppression	Cancer Discovery	Article	2018	689
6	Riquelme, Erick	Tumor Microbiome Diversity and Composition Influence Pancreatic Cancer Outcomes	Cell	Article	2019	679
7	Ahn, Jiyoung	Human Gut Microbiome and Risk for Colorectal Cancer	Journal Of The National Cancer Institute	Article	2013	654
8	Avgerinos, Konstantinos, I	Obesity and cancer risk: Emerging biological mechanisms and perspectives	Metabolism-Clinical and Experimental	Review	2019	613
9	Bartfeld, Sina	*In Vitro* Expansion of Human Gastric Epithelial Stem Cells and Their Responses to Bacterial Infection	Gastroenterology	Article	2015	528
10	Johnson, Amy R.	The inflammation highway: metabolism accelerates inflammatory traffic in obesity	Immunological Reviews	Review	2012	440

### Keyword visualization analysis

**Figure 8:**
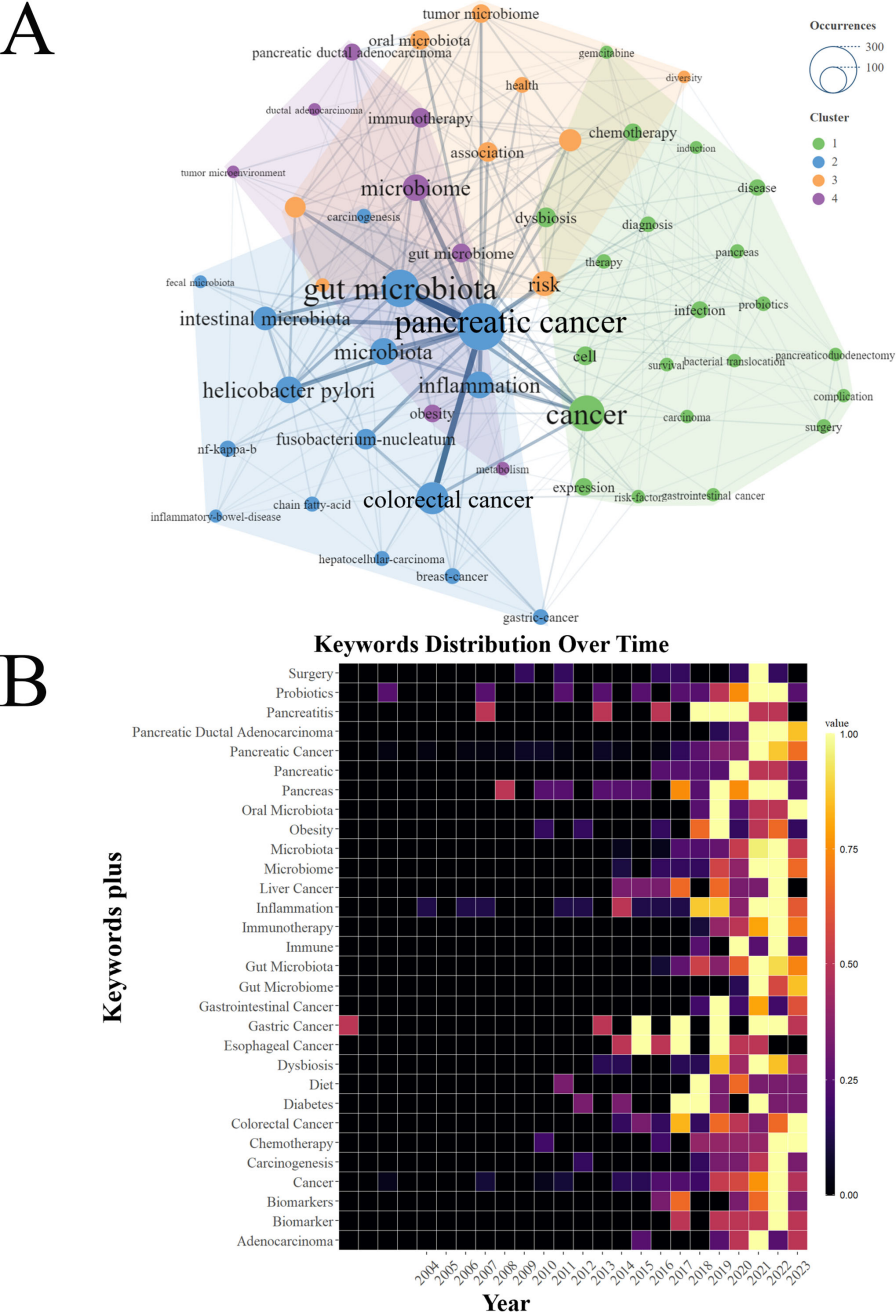
Keywords clustering and heatmap. **(A)** Clustering of the top 54 keywords with the highest number of occurrences by CiteSpace. **(B)** Timeline Visualization of Keywords Heatmap based on R software.

Keyword clustering forms interconnected networks of keywords with similar research themes. Each keyword cluster usually consists of one or more keywords that have a close relationship to one another, which was identified by header words that appear at high frequencies in the article. The keywords analysis of the 763 publications was performed by VOSviewer with a set threshold of 20. Among the top 54 keywords, four keywords clusters were developed according to keyword co-occurrence as shown in **Figure 8A**. The cluster consisting of “pancreatic cancer”, “gut microbiota”, “cancer”, “chemotherapy”, “microbiome”, “immunotherapy”, “risk” and “tumor microbiome” exhibited the highest frequency of occurrence (**Figure 8A**). In addition, the heat map of keywords based on Bibliometrix package revealed the distribution of research focus on evolution over time (**Figure 8B**). Notably, similar to the analysis of the keywords clustering, keywords of “pancreatic ductal adenocarcinoma”, “microbiome”, “chemotherapy”, “immunotherapy”, “gut microbiome” and “chemotherapy” have occurred frequently in recent years.

### Research trend topics analysis

**Figure 7C** presents the top 20 references exhibiting the strongest citation bursts in the field of PC and GM research between 2001 and 2023. The red bars indicate the time spans during which the citation bursts were sustained. Among these, the publication(DOI: 10.18632/oncotarget.3109) with the strongest citation burst (strength: 15.59) was by Kei Mitsuhashi et al., which began in 2017 and ended in 2020, focusing on the role of Fusobacterium species in PC (Mitsuhashi et al., 2015). The second strongest burst (strength: 12.45) was attributed to a study by Marie Vétizou et al. (DOI: 10.1126/science.aad1329), also spanning 2017–2020, which explored the influence of GM on anticancer immunotherapy (Vetizou et al., 2015). The third strongest citation burst (strength: 12.05) was for a paper(DOI: 10.1126/science.aac4255) by Ayelet Sivan et al., active from 2017 to 2020, investigating the contribution of commensal Bifidobacterium to antitumor immunity (Sivan et al., 2015). The keyword “immunotherapy” emerged as one of the most recent terms with strong citation bursts, suggesting it represents a current research hotspot in this area.

**Figure 9:**
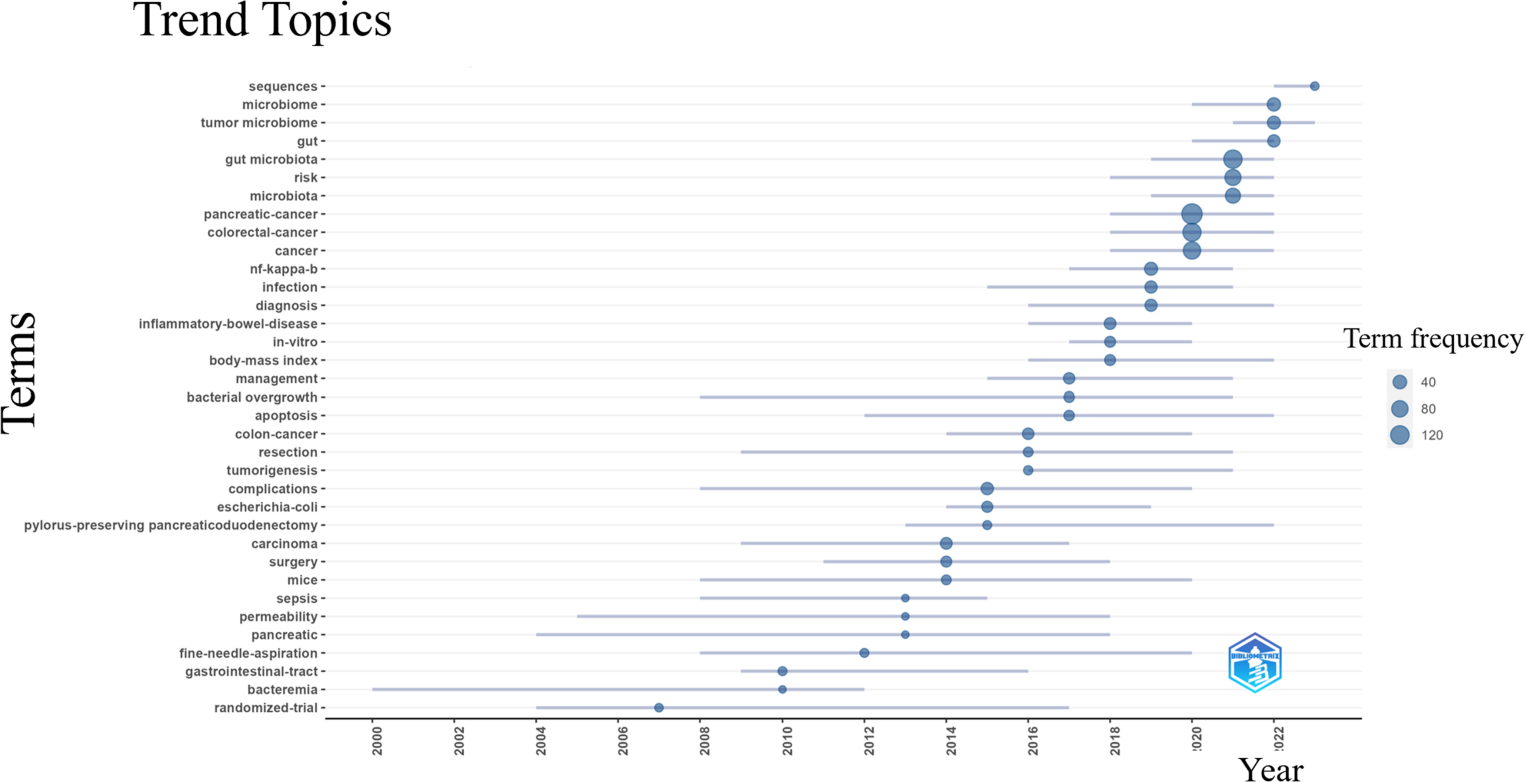
Trend topics over time based on R software. The onset of a blue line is defined by the first occurrence of a keyword, and the circle size is proportional to its frequency. The x-axis presents the year, and the y-axis shows the keywords.

**Figure 9** illustrates the evolution of global research trends. The blue line’s starting point marks the first appearance of a keyword, with circle size correlating to the frequency of its occurrence. The examination of the research trend topics from 2000 to 2023 identified “sequencing”, “microbiome”, “tumor microbiome”, “gut” as the research frontiers for the upcoming years.

## Discussion

In recent years, an increasing number of studies have explored the relationship between GM and PC, particularly in the last decade. Notably, some researches have shown a close link between GM dysfunction and PC (Pushalkar et al., 2018; Riquelme et al., 2019; da Cruz et al., 2025). This bibliometric analysis provided a comprehensive mapping of the knowledge structure and evolutionary trends in the research linking the GM to PC by analyzing highly productive countries, institutions, core journals, core literature, key authors, keyword clusters, and emerging research topics and future directions.

In this study, a total of 763 publications from January 1, 2000, to November 18, 2023, were analyzed to delineate research trends and identify potential future hotspots in this field over the past 23 years. The results indicate that interest in the association between GM and PC has steadily increased since 2014. The significant rise in literature over the last decade reflects a growing recognition of the impact of gut microbiota on PC. Regarding the country analysis, studies from 63 countries/regions were enrolled in this study. The analysis identified the USA and China as the two leading countries in this field, together accounting for 55.99% of all research (**Table 5**). The USA maintained a commanding lead over all other countries in total publications, H-index, and total citations, leading by a wide margin. Obviously, the USA’s status as the undisputed leader in this domain is likely underpinned by its robust research ecosystem, characterized by stable funding, deep expertise, and significant human capital. A striking contrast is presented by China, as a developing country, while the total publication volume and H-index were far ahead of other developed countries to rank second, but the average number of cited articles was relatively low. This phenomenon highlights the urgent need for increased investment and emphasis on fundamental science and original research. Additionally, institutions between countries should strengthen their cooperation, especially in developing countries. Although the USA and China had the closest interaction in the country collaboration, there was little close cooperation and communication among academic institutions in various nations (**Figure 4B**), with the majority of institutional cooperation was only within countries.

**Table 5:** The top 10 countries/regions with the highest productivity.

Rank	Country	Publications n(%)	Total citations	Average citations	Collaborative centrality
1	USA	259 (33.96%)	18736	72.34	0.22
2	China	168 (22.03%)	3895	23.18	0.05
3	Japan	68 (8.91%)	1842	27.09	0.05
4	Italy	67 (8.79%)	2667	39.81	0.16
5	Germany	51 (6.68%)	2173	42.61	0.11
6	United Kingdom	48 (6.29%)	1780	37.08	0.11
7	France	35 (4.59%)	3816	109.03	0.13
8	Spain	31 (4.07%)	783	25.26	0.03
9	Canada	30 (3.93%)	1955	65.17	0.05
10	Sweden	28 (3.67%)	2165	77.32	0.04

Regarding the institutional analysis, the ten leading organizations were distributed across four countries: the USA, China, Sweden, and Italy (**Table 1**). Among them, more than half of the top ten funded institutions came from the USA. Notably, there were 3 institutions from China in the top ten, including Zhejiang University, Shanghai Jiao Tong University and Peking Union Medical College. This reality further underscores the profound impact of economic investment and capacity on scientific advancement. Interestingly, although National Cancer Institute only ranked 10th in total papers, it ranked third in terms of average number of citations, which demonstrated that focusing on cutting-edge and major research topics yields highly innovative and influential outcomes. The multi-perspective analysis not only uncovered dense collaborative networks among institutions but also provides a valuable reference for researchers seeking suitable partners.

Regarding in the core journal analysis, those top ten journals published 18.48% of relevant articles, categorized into gastrointestinal cancer journals, general interest publications, and microbiological journals (**Table 3**). Notably, gastrointestinal cancer journals published the most articles, highlighting the gut microbiota’s significance in pancreatic cancer research. Analysis of the top 10 productive authors, Dominique S. Michaud (Brown University) was the most prolific author, contributing 160 publications, ahead of Florencia McAllister (88) and George Miller (76). Regarding scholarly impact, Richard B. Hayes and Jiyoung Ahn shared the highest citation counts (total: 1,464; average: 292.8). Hayes alone attained the highest H-index of 100. Based on this analysis, we can infer that research quality is a key driver of professional citation and engagement.

The top 10 core literatures with the highest citations on the association between GM and PC in **Table 4**. In this field, the first most cited article was written by Gopalakrishnan et al (Kokol, 2021). This article established that the structure and function of gut microbiota significantly affected outcomes of PD-1 inhibitor therapy, uncovering the microbiome’s impact on immune checkpoint blockade and paving the way for subsequent research into gut microbiota in PC The second most cited article was a review by McGuckin et al (Ge et al., 2022). This review focused on the interactions between intestinal pathogens and mucins, and the strategies pathogens employ to disrupt and circumvent the mucosal barrier, offering key insights for future investigations into the GM-PC mechanism. Notably, studies conducted by Pushalkar et al (Chen H. et al., 2023). dedicated to investigating the microbiome’s influence on development and PC prognosis. However, the exact role of the GM in PC pathogenesis remains unclear. Based on this analysis, we can infer that the current research focuses primarily on three themes: 1) how the gut microbiota influences immune regulation; 2) the mechanisms by which the gut microbiota contributes to tumorigenesis; and 3) the interactions among the microbiota, metabolism and cancer.

To better investigate and describe the evolution of research topics and frontiers on the link between GM and PC, this study employed timeline visualizations of co-cited references and keywords. These visualizations, particularly the timeline view of co-cited references shown in **Figures 7B** and **8B**, intuitively depict the temporal dynamics and shifts in research focus. The timeline view of co-cited references reveals a clear shift in research focus over time (**Figure 7B**). Recent studies have centered primarily on two areas: anticancer and metabolic pathway. Through the timeline visualizations of keywords and theme trend and thematic map, we found that the keywords “Gut Microbiota”, “Pancreatic Cancer”, “Cancer”, “Sequence” “Microbiome” and “Tumor Microbiome” may be hot research topics for the future.

Although the association between GM and PC has not been fully elucidated, considerable progress has been achieved in mechanistic research, paving the way for a deeper understanding. For one thing, Guo et al (Huang et al., 2023). found that the tumorigenicity of PDAC xenograft was higher in microbiota-intact mice than that in microbiota-depleted mice. Similarly, the study of Thomas et al (Thomas et al., 2018). demonstrated that complete PC mouse model was tended to develop carcinogenesis, compared with intestinal aseptic PC mouse model after the ABX cocktail therapy. These results indicated that the underlying reason may be related to the suppression of the host’s immune regulation by the gut microbiota. And, chronic inflammation in pancreatic tissue can trigger KRAS mutations in pancreatic endocrine cells ultimately leading to pancreatic epithelial tumors and PDAC, which was reported by Gidecel Friedlander et al (Gopalakrishnan et al., 2018). and Guerra et al (McGuckin et al., 2011). For another, established research demonstrated that specific microbes such as Bifidobacterium, Collinsella aerofaciens and Enterococcus faecalis can suppress regulatory T cells to enhance immune responses during cancer therapy (Zhang et al., 2020). Listeria monocytogenes was identified to promote the repolarization of macrophages from the immunosuppressive M2 phenotype to the antitumor M1 phenotype. Meanwhile, probiotics have been reported to inhibit PC development by producing anti-tumor mediator or enhancing the apoptosis of tumor cells (Kita et al., 2020; Konishi et al., 2021). Besides that, intestinal microbial may enhance the efficacy of conventional chemotherapy through drug metabolism, biological transformation. In the study conducted by Nicola Gaglian et al (Tintelnot et al., 2023). the microbiota-derived tryptophan metabolite indole-3-acetic acid (3-IAA) was enriched in patients who respond positively to chemotherapy through shotgun metagenomic sequencing and metabolomic screening. Simultaneously, fecal microbiota transplantation(FMT)was observed to increase the efficacy of chemotherapy in humanized gnotobiotic mouse models of PDAC, one of which may be involved in downregulation of the reactive oxygen species (ROS)-degrading enzymes (Riquelme et al., 2019). By searching clinical trial registration websites, we found that a relevant clinical trial NCT04975217(Fecal Microbial Transplants for the Treatment of Pancreatic Cancer) is underway. This study is focusing on the safety, tolerability, and feasibility of FMT in resectable PDAC patients, which is sponsored by M.D. Anderson Cancer Center.

This study has several limitations. Firstly, it only included publications from the Web of Science, a widely recognized database, which may have excluded some relevant works not indexed there. Actually, we previously considered merging data from multiple databases, such as Scopus, Medline, and Cochrane, to enhance literature coverage, but encountered several significant challenges during implementation. For more comprehensive results, those databases could be adopted in further studies. Secondly, our study was restricted to publications in English, which may have resulted in the exclusion of non-English studies and consequently limited the breadth of our findings. Thirdly, the specific search terms used potentially introduced selection bias by failing to capture all pertinent literature. Fourth, in this study, review articles account for nearly half of the total, which may introduce bias into the citation analysis of keywords. Lastly, given the rapidly evolving nature of microbiome research, some important and landmark studies may have been omitted and some hotspots that researchers are working on that have not been proven feasible may be missed, resulting in some new hotspots not being included. Obviously, it is subject to the inherent time lag in the Web of Science indexing process. It is hoped that these limitations will be addressed in future research.

In conclusion, we conducted a bibliometric analysis to thoroughly investigate the relationship between GM and PC, providing an overview of the topic and identifying future research directions in this field. The association between GM and PC has attracted increasing research interest, prompting more comprehensive investigations into this relationship. Based on this bibliometric analysis, we have revealed that GM plays a pivotal role in both the pathogenesis of PC and its therapeutic interventions.

## Data Availability

The original contributions presented in the study are included in the article/supplementary material. Further inquiries can be directed to the corresponding author.
